# Silencing RIF1 decreases cell growth, migration and increases cisplatin sensitivity of human cervical cancer cells

**DOI:** 10.18632/oncotarget.22315

**Published:** 2017-11-06

**Authors:** Ying Mei, Chen Peng, Yong-Bin Liu, Jing Wang, Hong-Hao Zhou

**Affiliations:** ^1^ Department of Clinical Pharmacology, Xiangya Hospital, Central South University, Changsha 410008, P. R. China; ^2^ Institute of Clinical Pharmacology, Central South University, Hunan Key Laboratory of Pharmacogenetics, Changsha 410078, P. R. China; ^3^ Hunan Province Cooperation Innovation Center for Molecular Target New Drug Study, Hengyang 421001, P. R. China; ^4^ Department of music therapy, Sam Houston State University, Huntsville TX 77340, USA; ^5^ Xiangya school of medicine, Central South University, Changsha 410008, P. R. China

**Keywords:** cervical cancer, RIF1, cisplatin, cell cycle, drug resistance

## Abstract

Replication timing regulatory factor 1 (RIF1) plays an important role in DNA replication regulation, stem cell pluripotency and DNA repair pathway. However, little is known about the molecular mechanisms and physiological significance of RIF1 in cancer and chemotherapy efficacy. In this study, we found that RIF1 is upregulated in cervical cancer tissues compared with normal tissues both at mRNA and protein levels through online databases. RIF1 knockdown reduced cervical cancer cell growth, colony formation, migration and epithelial–mesenchymal transition (EMT) markers. Flow cytometry analysis indicated that RIF1 knockdown induced apoptosis and G2 cell cycle arrest. Furthermore, RIF1 knockdown increased cisplatin sensitivity, cisplatin-induced G2/M phase arrest, apoptosis and led to defects in DNA repair in a concentration-dependent manner. In terms of mechanism research, increased CDKN1A expression and Bax/Bcl-2/caspase-3 signaling pathway might be involved in the G2/M phase arrest and increased apoptosis in RIF1-silenced cervical cancer cells. Thus, these findings indicate that RIF1 knockdown prior to chemotherapy may be a potential effective therapeutic strategy for cervical cancer.

## INTRODUCTION

Despite advances in diagnosis and therapeutic technologies, cervical cancer remains the major cause of death in developing countries and is the fourth most common cancer in female worldwide [1, 2]. Cisplatin (DDP) -based chemotherapy is a standard strategy for cervical cancer, while drug resistance remains a challenge [3–5]. Complex mechanisms are involved in platinum resistance, including decreased apoptosis, increased DNA repair capability and aberrant signaling pathway [3, 6].

RIF1 (replication timing regulatory factor 1) was originally identified in budding yeast as RAP1 interacting factor 1 with an essential role in telomere length maintenance [7]. Recently, RIF1 has been reported to play pivotal roles in the replication timing regulation and DNA damage response in fission yeast, budding yeast, and in mammals [8–12]. In human cells, RIF1 was found to be recruited to DNA double-strand breaks (DSBs) and acting downstream of the ATM/53BP1 to inhibit the 5’ end resection of broken DNA ends, facilitating non-homologous end joining (NHEJ) repair and restraining homologous recombination (HR) pathway [13, 14]. A latest paper indicated that depletion of endogenous RIF1 results in reduced DNA synthesis, indicating that RIF1 may regulate replication positively [15].

RIF1 plays an important role in stem cell pluripotency, DNA replication regulation and DNA repair pathway [12, 16, 17]. As far as we know, one paper reported that RIF1 was overexpressed in human breast tumors, and its expression status was positively correlated with differentiation degrees. Depletion of RIF1 sensitizes MCF7 cells to camptothecin (CPT) treatment [18]. Since then little is known about the role of RIF1 in cancer progression and chemotherapy response. And the function of RIF1 in cervical cancer has not been directly investigated too. In this study, we explored the role of RIF1 in cervical cancer cell growth, migration and platinum-based chemotherapy. We also investigated the underlying mechanisms and making RIF1 a promising therapeutic target.

## RESULTS

### RIF1 is overexpressed in human cervical carcinoma tissues

**Figure 1:**
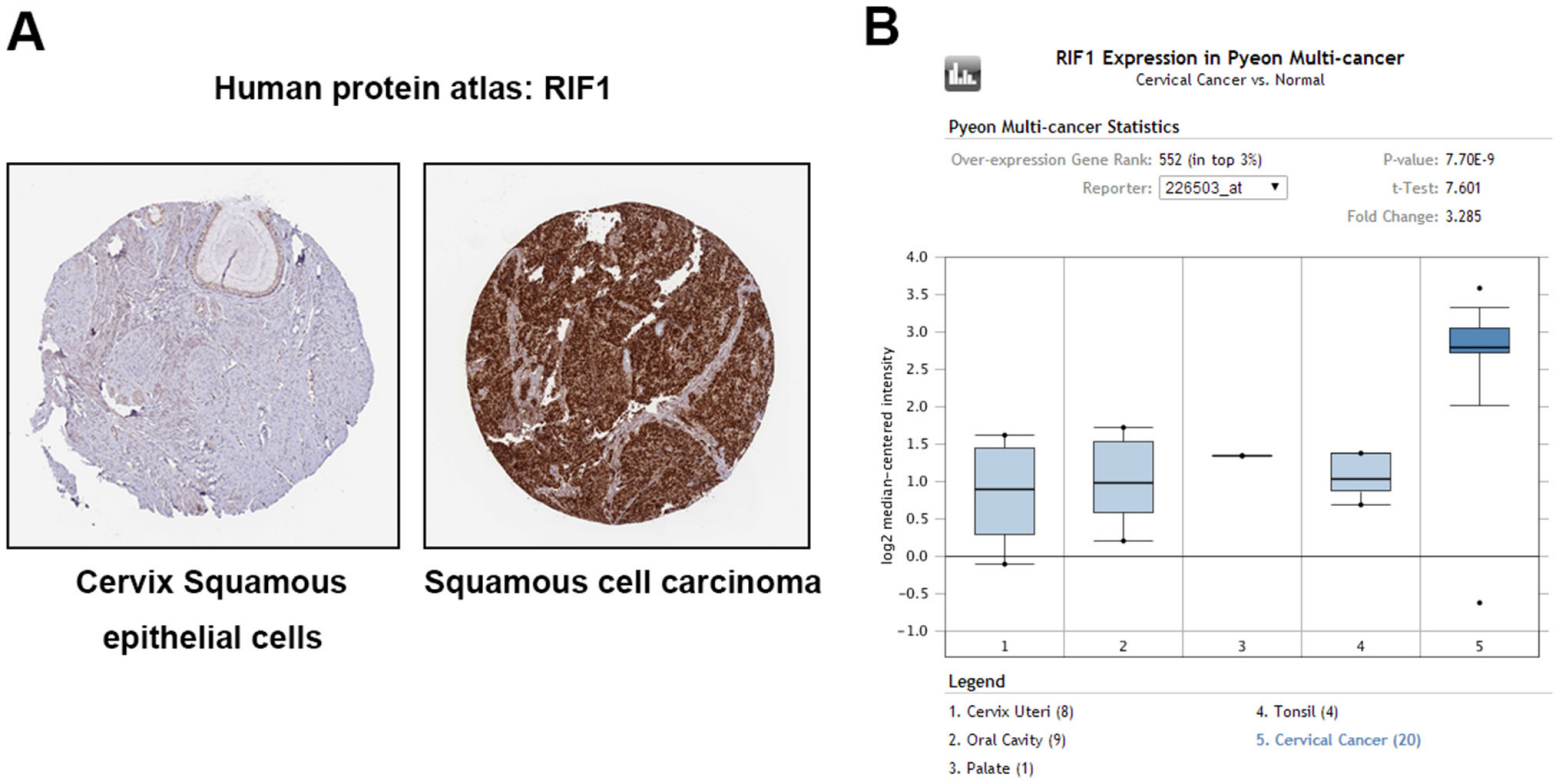
RIF1 is upregulated in human cervical carcinoma specimens **(A)** RIF1 expression in normal cervical tissue and cervical carcinoma specimens. Images were taken from the Human Protein Atlas (http://www.proteinatlas.org) online database. **(B)** Oncomine data indicating that RIF1 expression is overexpressed in cervical cancer tissues compared with normal tissues. (P= 7.5E-9).

To determine the clinical relevance of RIF1 expression in cervical carcinoma, we first analyzed the RIF1 protein expression in clinical tissue specimens from the human protein atlas (www.proteinatlas.org). We found that RIF1 had the positive strong expression in squamous cell carcinoma, and negative weak expression in normal cervix squamous epithelial cells (Figure 1A). Consistently, RIF1 mRNA level was significantly higher in cervical cancer tissues than that in normal tissues (P= 7.5E-9) in Pyeon Multi-cancer database (www.oncomine.org) (Figure 1B).

### Knockdown of RIF1 reduces cell proliferation and colony formation

**Figure 2:**
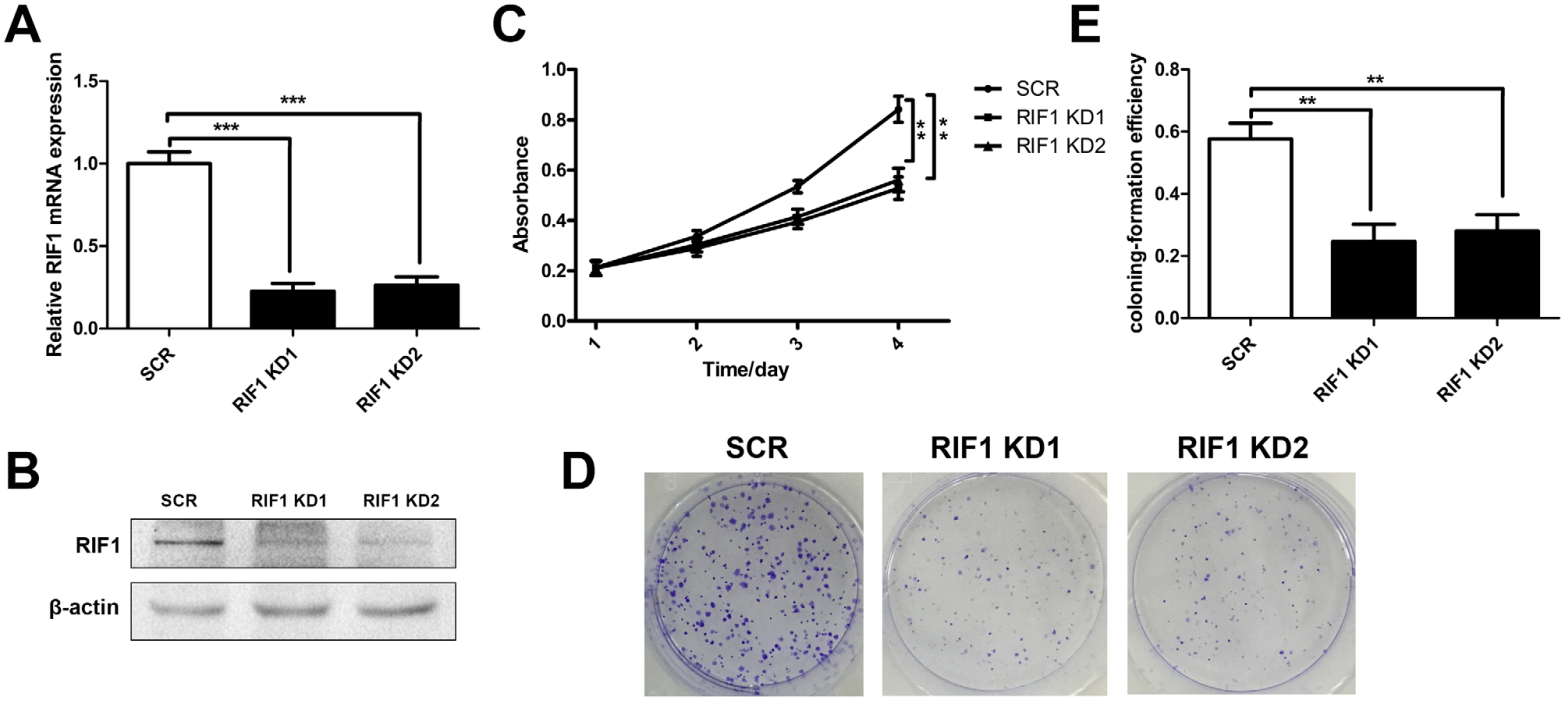
Knockdown of RIF1 inhibited the proliferation of cervical cancer cells (**A** and **B**) The silencing effect of the shRNAs to RIF1 (RIF1 knockdown, RIF1 KD) was confirmed by RT-qPCR and Western blot and in HeLa cells. **(C)** Proliferation of HeLa cells transfected with shRNAs to RIF1 or scrambled control vector (SCR). **(D)** Colony formation assay of RIF1 silenced HeLa cells compared with scrambled control. **(E)** Quantification of Colony formation efficiency. Date were shown as mean ± SD of three independent experiments. ^*^ P<0.05, ^**^ P<0.01, ^***^ P<0.001.

We knocked down RIF1 in HeLa cell lines through shRNA transfection, mRNA and protein levels were significantly decreased in shRIF1 transfectants which determined by real time RT-PCR and Western blot analyses (Figure 2A and 2B). Cell proliferation was calculated via MTS and colony formation assays were performed after knockdown of RIF1 by shRNAs. As shown in Figure 2C, MTS assays demonstrated that knockdown of RIF1 could significantly suppress the proliferation activity of the HeLa cells in contrast with scrambled control groups at 4 days after transfection (^**^ p < 0.01). In addition, colony formation assay indicated that silencing of RIF1 drastically decreased the number of colonies formed compared with scrambled control groups (Figure 2D and 2E).

### Downregulation of RIF1 inhibited cell cycle progression and promoted cell apoptosis through CDKN1A and Bax/Bcl-2/caspase-3 pathway

**Figure 3:**
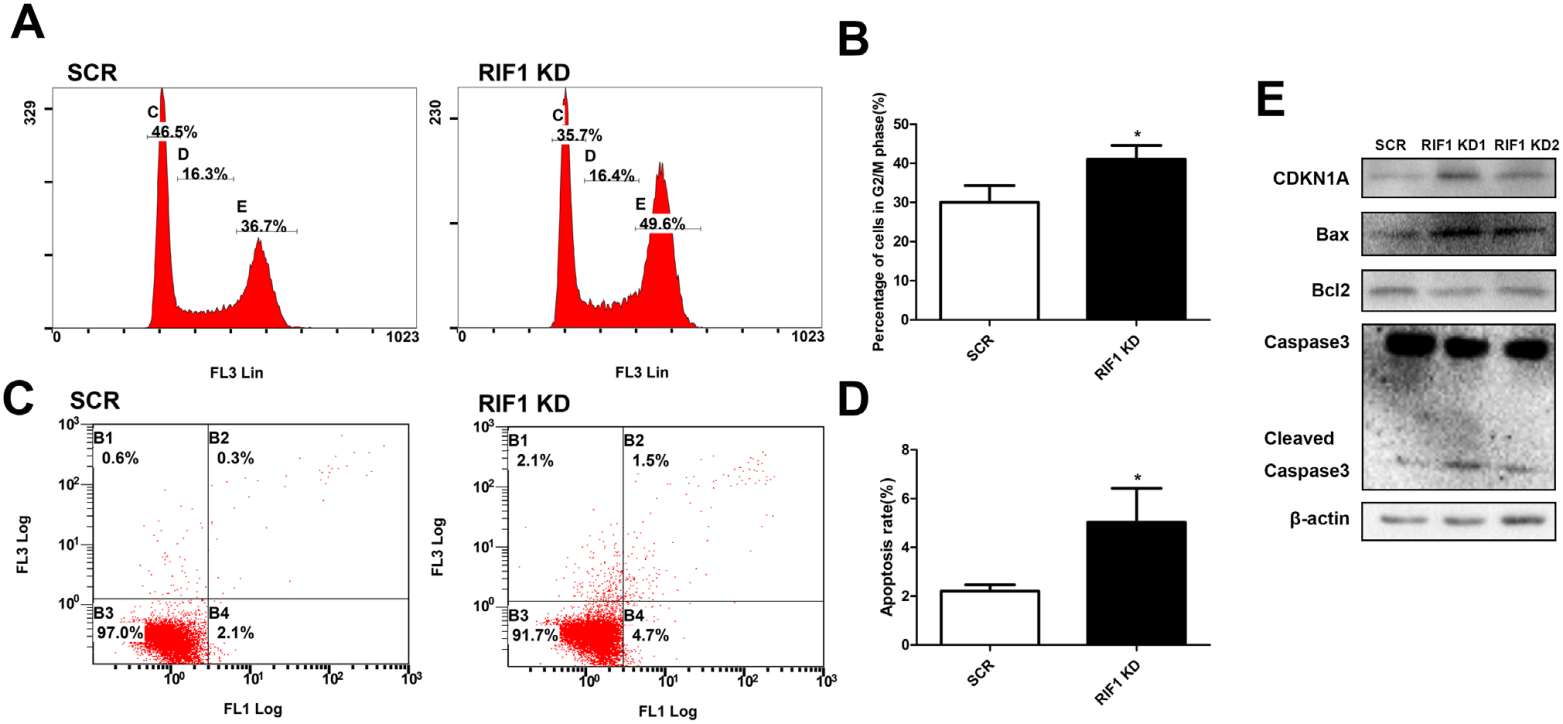
Effect of RIF1 on cervical cancer cell cycle progression and apoptosis **(A, B)** Knockdown of RIF1 dramatically increased the G2/M phase fraction. **(C, D)** Annexin V-FITC/PI staining demonstrated that knockdown of RIF1 significantly enhanced the apoptosis rate compared with scrmbled control groups in HeLa cells. **(E)** Western blot analysis was performed to evaluate the protein expression of CDKN1A, Bax, Bcl2, Caspase3 and Cleaved Caspase3. Date were shown as mean ± SD of three independent experiments. ^*^ P<0.05 vs. control group.

To explore whether the repressive effect of RIF1 knockdown in the proliferation of cervical cancer cells was mediated by inhibiting cell cycle progression or promoting apoptosis, flow cytometry analysis was performed. As shown in Figure 3A and 3B, cell cycle analyses demonstrated that knockdown of RIF1 led to an obviously accumulation of cells in G2/M phase (^*^ p < 0.05), in comparison with control groups. We then examined the protein levels of CDKN1A (P21) which is a G2/M transition key regulator. After knockdown of RIF1 the expression of CDKN1A was greatly increased compared with the scrambled control groups, which indicated that RIF1 might exert its functions through CDKN1A-mediated G2/M cell cycle transition signaling pathway.

Since aberrant regulation of apoptosis is one of the contributing factors to cancer progression and development, we then examined the effects of RIF1 on the apoptosis of cervical cancer cells by Annexin V-FITC/PI staining assay. As shown in Figure 3C and 3D, the proportions of apoptotic cells with RIF1 knockdown were significantly increased compared with those in the scrambled control groups (^*^ p < 0.05). These results demonstrated that down-regulation of RIF1 could inhibit tumor cell proliferation via promoting apoptosis in cervical cancer.

To further illustrate the molecular mechanisms involved in the apoptosis resulting from RIF1 knockdown, we next tested the expression of Bax, Bcl-2 and caspase-3, which are important for cell apoptosis. We observed upregulation of cleaved Caspase-3 and Bax and downregulation of Bcl-2 (Figure 3E), indicating that an increase in the Bax/Bcl-2 ratio and activation of Caspase-3 might be involved in the apoptosis induced by RIF1 knockdown in HeLa cells.

### Downregulation of RIF1 decreased migration and EMT markers

**Figure 4:**
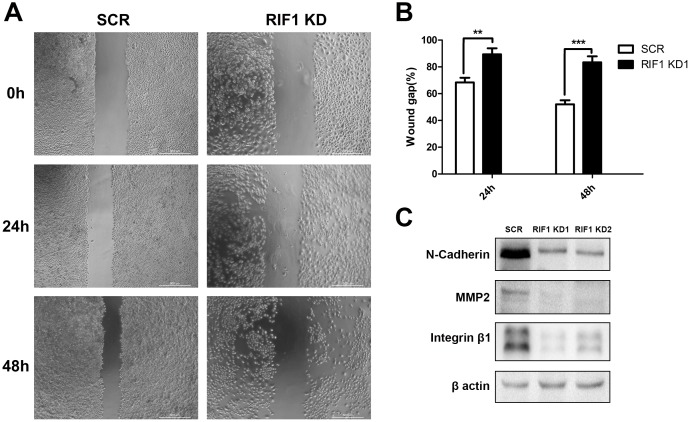
Downregulation of RIF1 decreased migration and EMT markers **(A)** Migration of HeLa cells with or without RIF1 knockdown was measured by wound healing assay. **(B)** Quantification of wound gap of RIF1 silenced HeLa cells at 24h and 48h after application of scratch wound. **(C)** Western blot analysis was performed to evaluate the protein expression of N-cadherin, MMP2 and Integrin-β1 in HeLa cells. Data were described as mean ±SD of three independent experiments. ^*^ P<0.05, ^**^ P<0.01, ^***^ P<0.001.

Cell migration was tested by scratch wound healing assay, RIF1 knockdown significantly decreased cell migration after 24 h and 48 h of incubation (Figure 4A and 4B). Epithelial-mesenchymal transition (EMT) plays a pivotal role in migration and metastasis of many kinds of cancers [19, 20]. Thus, we tested the expression of well-recognized EMT markers, such as N-cadherin, MMP2 and Integrin β1. As shown in Figure 4C, we found that the expression levels of N-cadherin, MMP2 and Integrin β1 were significantly decreased in RIF1 knockdown groups, suggesting that silencing of RIF1 might inhibit migration of cervical cancer cells by inhibiting the EMT signaling pathway.

### Effect of RIF1 knockdown on cellular response to cisplatin

**Figure 5:**
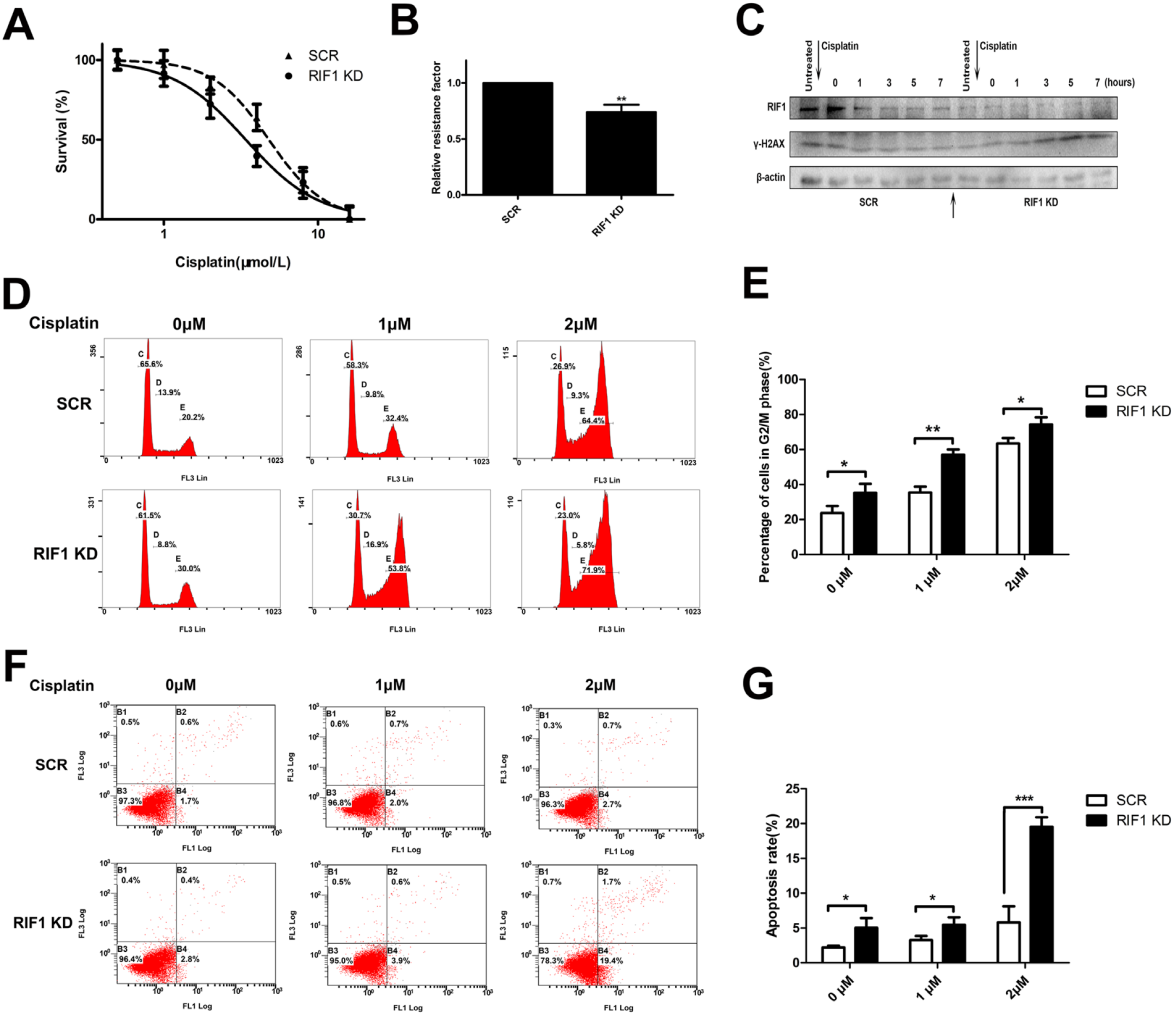
RIF1 knockdown increases cisplatin sensitivity, cisplatin-induced G2/M phase arrest, apoptosis and leads to defects in DNA repair **(A)** Concentration-dependent growth inhibition in response to cisplatin in RIF1-silenced and scrambled control HeLa cells. **(B)** Half maximal inhibitory concentration (IC50) was calculated by Graphpad 5 from 3 independent experiments. Relative resistance factor (RRF) of cisplatin was calculated by dividing the IC50 of the control cells and by that of the cells with RIF1 knockdown. **(C)** HeLa cells were treated with cisplatin for 1 h, washed free of cisplatin (0 time point) and then harvested at various time points. Total cell lysate was immunoblotted for γ-H2AX antibody, which is a marker for damaged DNA not repaired yet. **(D, E)** RIF1 knockdown increases cisplatin induced G2/M phase arrest in a concentration-dependent manner compared with scrambled control. **(F, G)** Annexin V/PI staining showed that RIF1 knockdown HeLa cells enhanced cisplatin-induced apoptosis rate compared with scrambled control in a concentration-dependent manner. Data were presented as means ± SD of three independent experiments. ^*^ P<0.05, ^**^ P<0.01, ^***^ P<0.001.

As RIF1 participates in cell cycle, apoptosis and DNA repair pathway, and all these signaling pathways are involved in the pharmacodynamics mechanism of platinum drugs, we investigated the involvement of RIF1 in sensitivity to cisplatin in cervical cancer. To confirm whether RIF1 affects the efficacy of cisplatin, HeLa cells were subjected to MTS assay in the presence or absence of various concentrations of cisplatin. As illustrated in Figure 5A and 5B, RIF1-silenced HeLa cells became more sensitive to cisplatin with about 30% decrease in relative resistance factor.

### Knockdown of RIF1 increases cisplatin-induced apoptosis and G2/M phase arrest

It is widely considered that DNA is the major target of cisplatin [21], resulting in DNA damage and further inducing apoptosis, which is one of the major action of mechanisms of cisplatin [22]. Hence we further examined whether RIF1 knockdown increased cisplatin-induced apoptosis. As shown in Figure 5F and 5G, the apoptosis rate was increased in RIF1-silenced HeLa cells after cisplatin treatment in a concentration-dependent manner.

Moreover, as shown in 5D and 5E, cisplatin treatment led to G2/M phase cell cycle arrest. Interestingly, RIF1 knockdown dramatically increased the percentage of G2/M phase cells in a concentrate-dependent manner compared with scrambled control cells with cisplatin treatment. These results indicate that RIF1 contributes to cellular response to cisplatin partly via regulating cell apoptosis and G2/M cell cycle arrest.

### Effect of RIF1 knockdown on DNA repair

Increased DNA repair is widely considered as one of the major mechanisms of platinum resistance [6] and RIF1 has been reported to act downstream of the ATM/53BP1, promoting NHEJ repair [14]. So we explored whether RIF1 affected cervical cancer cellular DNA repair capacity upon cisplatin treatment. The time course of the expression of γ-h2AX (a marker of DSB severity) after cisplatin treatment was analyzed by western blot analysis. RIF1 knockdown and scrambled control HeLa cells were treated with cisplatin for 1 h and then washed free of the drug. The expression of γ-h2AX was significantly decreased as time went on in control HeLa cells. However, in RIF1 knockdown cells, γ-H2AX levels were increased along the time and reached the highest value till 9 h after cisplatin removal (Figure 5C). Persistence of high γ-H2AX levels in RIF1-silenced cells suggested that RIF1 is pivotal for efficient DNA repair upon cisplatin treatment.

## DISCUSSION

RIF1 has been illustrated to play a significant role in DNA repair and DNA replication regulation pathway, but little is known about the role of RIF1 in cancer development and chemotherapy response. RIF1 is upregulated at both mRNA and protein levels in clinical cervical cancer specimens, indicating its clinical significance. Based on the intriguing findings from the clinical samples, we then explored the underlying molecular mechanisms in cell models. We found that knockdown of RIF1 not only inhibited cell proliferation but also resulted in G2/M cell cycle arrest and promoted cell apoptosis. To explore the underlying mechanisms, we examined the expression levels of CDKN1A, Bax, Bcl-2 and caspase-3. We found that the expression of CDKN1A, G2/M transition key regulator, was increased. Simultaneously, we observed upregulation of Bax, activation of Caspase3 and downregulation of Bcl-2. These results suggest that increased CDKN1A expression and Bax/Bcl-2/caspase-3 signaling pathway might be involved in the G2/M phase arrest and increased apoptosis in RIF1-silenced cervical cancer cells.

Additionally, we discovered that silencing of RIF1 greatly inhibited migration of cervical cancer cells, and found that downregulation of RIF1 significantly decreased the expression of EMT markers, such as N-cadherin, MMP2 and Integrin-β1. These findings suggest that RIF1 plays a pivotal role in cervical cancer metastasis.

A previous paper has reported that depletion of RIF1 sensitizes MCF7 cells to CPT treatment, so we explored that whether RIF1 affects the efficacy of cisplatin [18]. We found that downregulation of RIF1 dramatically enhanced the chemosensitivity to cisplatin. Consistently, we discovered that the percentage of cells in apoptosis and G2/M cell cycle arrest for RIF1-silenced HeLa cells treated with cisplatin was significantly higher than for control cells.

Apart from regulating apoptosis and cell cycle progression, RIF1 plays a significant role in NHEJ repair [14]. On the one hand, compared with error-free HR repair pathway, the NHEJ repair is error-prone, leading to mutation accumulation and carcinogenesis. On the other hand, high expression of RIF1 in cancer cells was hypothesized to result in increased DSB repair and confer drug resistance [23]. In this study, knockdown of RIF1 inhibited DSB repair and cell survival upon cisplatin treatment. Understanding the mechanisms behind chemoresistance may allow stratification of treatment selection based on RIF1 expression levels, as well as potentially allowing molecular targeting to increase chemosensitivity [24].

In summary, our novel findings show that downregulation of RIF1 could not only inhibit cancer cells proliferation and migration but also enhance cisplatin-induced growth inhibition, G2/M cell cycle arrest, apoptosis and DNA damage in cervical cancer, suggesting that RIF1 may be a novel strategy for cervical cancer therapy.

## MATERIALS AND METHODS

### Cell culture and cell lines

Human cervical cancer cell lines, HeLa, was purchased from the cell banks of the Shanghai Institutes of Biological Sciences (Shanghai, China) and was tested and authenticated before use. The cells were cultured in RPMI-1640 medium (Corning Inc., Corning, NY, USA) containing 10% fetal bovine serum (FBS).

### ShRNA transfection

Two shRNA sequences targeting human RIF1 cDNA was synthesized by GenePharma: RIF1 KD1: 5’-GCCTTTGAGTTCCATCCAT-3’, KD2: 5’-AAGAGCATCTCAGGGTTTGCT-3’. The targeting sequence of scrambled control of shRNA vector is 5’-TTCTCCGAACGTGTCACGT-3’. Cells were transiently transfected with scrambled control or shRNAs using ViaFect™ transfection reagent (Promega) according to the protocol.

### Flow cytometric analysis

The apoptosis rates were detected using FITC Annexin V Apoptosis Detection Kit I (BD Biosciences, San Jose, CA, USA) following the instructions of the manufacturer’s instructions. Cell cycle was measured using the Cell Cycle and Apoptosis Analysis Kit (Beyotime Institution of Biotechnology, Shanghai, China) according to the protocol. Cells were analyzed by FC500 flow cytometry instrument equipped with CXP software. (Beckman Coulter, Bethesda, MA, USA).

### Western blotting

Western blotting was performed as described previously [25]. Briefly, for Western blotting, proteins were extracted using RIPA buffer (50 mM Tris-HCl, pH 7.4, 150 mM NaCl, 1% sodium deoxycholate, 0.1% SDS, 1mM Na2EDTA, 1% Triton X-100, 1 μg ml^-1^ leupeptin) supplemented with protease inhibitors and phosphatase inhibitors (Biotool). The lysate was centrifuged at 13000 rpm at 4°C for 15 min. The supernatants were collected and protein concentrations were measured using the BCA method. Proteins were then immunoblotted on the PVDF membrane by standard procedures. Antibodies against RIF1 were obtained from Bethyl Laboratories. Antibodies to Bax, Bcl2, N-Cadherin, MMP2, and Integrin-β1 were purchased from Cell Signaling Technology. Antibodies against caspase3 were obtained from Santa Cruz. Antibody to β-actin was purchased from Sigma-Aldrich and used as a loading control.

### RNA isolation and real-time quantitative PCR

Total RNA was isolated from cultured cells by TRIzol reagent (Invitrogen, Grand Island, NY, USA) Reverse transcription was performed by PrimeScript RT reagent Kit With gDNA Eraser (TaKaRa), and the quantitative RT-PCR was performed using SYBR Premix DimerEraser (Perfect Real Time) kit (TaKaRa) on the Roche LightCycler480 (Roche, San Francisco, CA, USA). Data were analyzed by the -2^ΔΔct^ method and the expression of β-actin was used as normalization control.

### Cell viability analysis

RIF1-silenced cells were seeded in 96-well plates at a density of 3 × 10^4^ cells in 100 μl medium and incubated with cisplatin (Sigma) for 48 hours. Cell viability was evaluated by MTS approach according to the instructions for Cell Titer 96 Aqueous-One-Solution Cell Proliferation Assay kit (MTS). The IC50 was calculated from the dose-response curves using GraphPad Prism 5.0 program (GraphPad Software, Inc.).

### Analysis of RIF1 mRNA and protein expression in human cervical cancer

RIF1 protein expression in human cervical cancer tissues and normal tissues was determined from the human protein atlas (www.proteinatlas.org). RIF1 gene expression was determined through data from Oncomine database (www.oncomine.org).

### Colony formation assay and wound healing assay

The transfected HeLa cells were plated in 6-well plates at 500 cells per well and cultured for 8 days. The colonies were fixed with 4% paraformaldehyde for 30 minutes and then stained with 1% crystal violet (Beyotime Institute of Biotechnology, Shanghai, China) for 15 minutes.

For wound healing assay, cells were seeded in six-well plates and were transfected with different plasmids. Upon the cell reached a confluent monolayer, a scratch wound was made on the cell monolayer using a sterile pipette tip. The cells were then washed with phosphate-buffered saline (PBS) to remove the debris and then replaced with a medium containing 1% FBS. The images was photographed at 0 h, 24 h and 48 h after woundind using a microscope.

### Statistical analysis

All data were described as mean ±standard deviation (SD). Two-tailed Student’s t test was used to derive the significance between two groups for continuous variables. Statistical analysis was conducted using SPSS 18.0 (SPSS Inc.). *P*≤0.05 was considered statistically significant.

## References

[1] Siegel RL, Miller KD, Jemal A (2017). Cancer Statistics, 2017. CA Cancer J Clin.

[2] Li Y, Cui N, Zheng PS, Yang WT (2017). BMX/Etk promotes cell proliferation and tumorigenicity of cervical cancer cells through PI3K/AKT/mTOR and STAT3 pathways. Oncotarget.

[3] Yu WK, Wang Z, Fong CC, Liu D, Yip TC, Au SK, Zhu G, Yang M (2017). Chemoresistant lung cancer stem cells display high DNA repair capability to remove cisplatin-DNA damage. Br J Pharmacol.

[4] Feng Y, Zou W, Hu C, Li G, Zhou S, He Y, Ma F, Deng C, Sun L (2017). Modulation of CASC2/miR-21/PTEN pathway sensitizes cervical cancer to cisplatin. Arch Biochem Biophys.

[5] Liang S, Peng X, Li X, Yang P, Xie L, Li Y, Du C, Zhang G (2015). Silencing of CXCR4 sensitizes triple-negative breast cancer cells to cisplatin. Oncotarget.

[6] Stewart DJ (2007). Mechanisms of resistance to cisplatin and carboplatin. Crit Rev Oncol Hematol.

[7] Hardy CF, Sussel L, Shore D (1992). A RAP1-interacting protein involved in transcriptional silencing and telomere length regulation. Genes Dev.

[8] Hayano M, Kanoh Y, Matsumoto S, Renard-Guillet C, Shirahige K, Masai H (2012). Rif1 is a global regulator of timing of replication origin firing in fission yeast. Genes Dev.

[9] Yamazaki S, Ishii A, Kanoh Y, Oda M, Nishito Y, Masai H (2012). Rif1 regulates the replication timing domains on the human genome. EMBO J.

[10] Peace JM, Ter-Zakarian A, Aparicio OM (2014). Rif1 regulates initiation timing of late replication origins throughout the S. cerevisiae genome. PLoS One.

[11] Kanoh Y, Matsumoto S, Fukatsu R, Kakusho N, Kono N, Renard-Guillet C, Masuda K, Iida K, Nagasawa K, Shirahige K, Masai H (2015). Rif1 binds to G quadruplexes and suppresses replication over long distances. Nat Struct Mol Biol.

[12] Foti R, Gnan S, Cornacchia D, Dileep V, Bulut-Karslioglu A, Diehl S, Buness A, Klein FA, Huber W, Johnstone E, Loos R, Bertone P, Gilbert DM (2016). Nuclear Architecture Organized by Rif1 Underpins the Replication-Timing Program. Mol Cell.

[13] Silverman J, Takai H, Buonomo SB, Eisenhaber F, de Lange T (2004). Human Rif1, ortholog of a yeast telomeric protein, is regulated by ATM and 53BP1 and functions in the S-phase checkpoint. Genes Dev.

[14] Zimmermann M, Lottersberger F, Buonomo SB, Sfeir A, de Lange T (2013). 53BP1 regulates DSB repair using Rif1 to control 5′ end resection. Science.

[15] Hiraga SI, Ly T, Garzón J, Hořejší Z, Ohkubo YN, Endo A, Obuse C, Boulton SJ, Lamond AI, Donaldson AD (2017). Human RIF1 and protein phosphatase 1 stimulate DNA replication origin licensing but suppress origin activation. EMBO Rep.

[16] Wang J, Rao S, Chu J, Shen X, Levasseur DN, Theunissen TW, Orkin SH (2006). A protein interaction network for pluripotency of embryonic stem cells. Nature.

[17] Xu D, Muniandy P, Leo E, Yin J, Thangavel S, Shen X, Ii M, Agama K, Guo R, Fox D, Meetei AR, Wilson L, Nguyen H (2010). Rif1 provides a new DNA-binding interface for the Bloom syndrome complex to maintain normal replication. EMBO J.

[18] Wang H, Zhao A, Chen L, Zhong X, Liao J, Gao M, Cai M, Lee DH, Li J, Chowdhury D, Yang YG, Pfeifer GP, Yen Y, Xu X (2009). Human RIF1 encodes an anti-apoptotic factor required for DNA repair. Carcinogenesis.

[19] Kang AR, An HT, Ko J, Kang S (2017). Ataxin-1 regulates epithelial-mesenchymal transition of cervical cancer cells. Oncotarget.

[20] Tian T, Li X, Hua Z, Ma J, Wu X, Liu Z, Chen H, Cui Z (2017). S100A7 promotes the migration, invasion and metastasis of human cervical cancer cells through epithelial-mesenchymal transition. Oncotarget.

[21] Yu WK, Wang Z, Fong CC, Liu D, Yip TC, Au SK, Zhu G, Yang M (2017). Chemoresistant lung cancer stem cells display high DNA repair capability to remove cisplatin-induced DNA damage. Br J Pharmacol.

[22] Wang D, Lippard SJ (2005). Cellular processing of platinum anticancer drugs. Nat Rev Drug Discov.

[23] Yin JY, Shen J, Dong ZZ, Huang Q, Zhong MZ, Feng DY, Zhou HH, Zhang JT, Liu ZQ (2011). Effect of eIF3a on response of lung cancer patients to platinum-based chemotherapy by regulating DNA repair. Clin Cancer Res.

[24] Yuan SS, Hou MF, Hsieh YC, Huang CY, Lee YC, Chen YJ, Lo S (2012). Role of MRE11 in cell proliferation, tumor invasion, and DNA repair in breast cancer. J Natl Cancer Inst.

[25] Samaeekia R, Adorno-Cruz V, Bockhorn J, Chang YF, Huang S, Prat A, Ha N, Kibria G, Huo D, Zheng H, Dalton R, Wang Y, Moskalenko GY, Liu H (2017). miR-206 Inhibits Stemness and Metastasis of Breast Cancer by Targeting MKL1/IL11 Pathway. Clin Cancer Res.

